# Comparative study of the effect of U100 laser and pneumatic ballistic combined with percutaneous transhepatic cholangioscopic lithotomy in the treatment of intra-and extrahepatic bile duct stones and its effect on liver function

**DOI:** 10.12669/pjms.38.6.5903

**Published:** 2022

**Authors:** Zhongliang Yin, Bin Shen

**Affiliations:** 1 Zhongliang Yin, Dept. of Surgery, Jiaxing Maternal and Child Health Hospital, Jiaxing, Zhejiang Province 314001, P.R. China; 2 Bin Shen, Dept. of Surgery, Jiaxing Traditional Chinese Medicine Hospital, Jiaxing, Zhejiang Province 314000, P.R. China

**Keywords:** U100 laser lithotripsy, Percutaneous transhepatic cholangioscopic lithotripsy, intra-and extrahepatic bile duct stones, Operation, Liver function

## Abstract

**Objectives:**

To compare the efficiency of U100 laser and pneumatic ballistics combined with percutaneous transhepatic cholangioscopic lithotripsy (PTCSL) in the treatment of intra-and extrahepatic bile duct stones and their effects on liver function.

**Methods:**

Medical records of 97 patients with intra-and extrahepatic bile duct stones treated in our hospital from May 2020 to May 2021 were selected for retrospective analysis. Of them, forty-three patients received pneumatic ballistic lithotripsy combined with PTCSL (Group-I), and 54 patients received U100 laser lithotripsy combined with PTCSL (Group-II). The therapeutic effects of the treatment in two groups and its effect on liver function were compared and analyzed.

**Results:**

There was no significant difference in the operation time and intraoperative bleeding (P>0.05) between the two groups. The postoperative pain duration and hospital stay of patients treated with U100 laser lithotripsy combined with PTCSL (Group-II) were shorter than those treated with pneumatic ballistic lithotripsy combined with PTCSL (Group-I), P<0.05. The biliary bleeding rate of patients in Group-II was lower (3.70%) than those in Group-I (16.27%, P<0.05), and the stone residue rate of patients in Group-II was also lower (1.85%) than those in Group-I (11.63%, P<0.05). The levels of total bilirubin (TBIL), alanine aminotransferase (ALT) and albumin (ALB) in Group-II patients were higher than in patients in Group-I.(P<0.05).

**Conclusion:**

Compared with pneumatic ballistics, U100 laser lithotripsy combined with PTCSL in the treatment of intra and extrahepatic bile duct stones has the advantages of less postoperative pain, shorter hospital stay, less biliary bleeding and stone residue, and less damage to liver function.

## INTRODUCTION

The high residual stone rate, high recurrence rate and high reoperation rate of intrahepatic and extrahepatic bile duct stones are still difficult problems in biliary surgery.^1^,^2^ The intrahepatic bile duct is curved, narrow and complex. Most intra-and extrahepatic bile duct stones are grouped and sporadic and can cause hepatobiliary stricture. These stones can also block the bile duct, which can easily cause recurrent cholangitis, resulting in fever, abdominal pain and other symptoms.^3^ Additionally, if biliary obstruction is not removed in time, it can lead to the accumulation of a large number of bacteria in the biliary tract, resulting in the continuous increase of biliary pressure, which promotes the diffusion of infectious substances in the biliary tract. Bacteria and toxins can enter the hepatic sinuses and arteries, leading to systemic infection and further complications, such as obstructive suppurative cholangitis and liver abscess that may endanger the lives of patients.^4^

The traditional and more conservative treatment in cases of intrahepatic and extrahepatic bile duct stones involves anti-inflammatory agents and liver protection. However, these methods have limited effectiveness, and can often result in surgery to treat intra-and extrahepatic bile duct stones. Percutaneous transhepatic cholangioscopic lithotripsy (PTCSL) is a common surgical procedure which can effectively remove these stones, relieve the obstruction, and promote unobstructed drainage.^5^

There are many lithotripsy methods used for the treatment of intra-and extrahepatic bile duct stones. Pneumatic ballistic lithotripsy is the most common and can effectively crush stone. However, it is associated with stone residues and is not that efficient in case of granulation tissue.^6^ U100 laser lithotripsy converts the laser power into shock waves which can “disintegrate” the stone from the center, causing less driving force. This reduces the damage to the bile duct and liver tissue.^7^ In recent years, the combination of U100 laser lithotripsy with percutaneous transhepatic cholangiolithotomy has been used in our hospital to treat patients with intra-and extrahepatic bile duct stones. The main goal of this study is to compare the efficiency of U100 laser and pneumatic ballistics combined with percutaneous transhepatic cholangioscopic lithotripsy in the treatment of intra-and extrahepatic bile duct stones and their effects on liver function.

##  METHODS

The records of 97 patients (51 males and 46 females) with intra-and extrahepatic bile duct stones, treated in our hospital from May 2020 to May 2021, were analyzed retrospectively. Of them, 43 patients that were treated with pneumatic ballistic lithotripsy combined with PTCSL comprised Group-I, and 54 patients that were treated with U100 laser lithotripsy combined with PTCSL were defined as Group-II.

### The inclusion criteria

Meets the diagnostic criteria of intra-and extrahepatic bile duct stones based on the results of the ultrasound, CT and other imaging examinations;^8^ Intrahepatic bile duct dilatation >4mm; No history of previous laparotomy or biliary surgery; Aware of the study and cooperate with informed consent.

###  Exclusion criteria

Severe basic diseases, organ dysfunction and malignant tumour; Contraindications of PTCSL; Incomplete medical records. The medical ethics Association of our college approved the study (No. JXFY-L20210311, Date: 2021-March-10).

Following anesthesia, the percutaneous transhepatic puncture path was determined according to the preoperative examination. The puncture point, was located in the abdominal wall of the lower right edge of the xiphoid process or between the right 8th~10th ribs. The percutaneous transhepatic puncture was completed using ultrasound real-time monitoring. After the puncture needle entered the target bile duct, the inner core was pulled out, and the bile was withdrawn. After the successful puncture was confirmed, the guide wire was injected into the puncture needle tube and the fistula was dilated through the 8F fascia expander in the direction of the guide wire.

After no bleeding was observed, the matching sheath sleeve was sleeved on the 16F or 18F expander and delivered to the target intrahepatic bile duct. The expander was pulled out and the sheath tube was left. One end of the sheath tube was in the intrahepatic bile duct and the other end was left in vitro to place the fistula between the intrahepatic bile duct and the outer surface of the body. The choledochoscope was placed into the target bile duct through this channel. Saline was flushed through, which allowed the small stones to flow out of the body or be removed with a blue net.

Pneumatic ballistic lithotripsy was done using pneumatic ballistic lithotripter (EMS SA CH21260 NYON, Switzerland). The air pressure was set to 2×100kPa, which broke up the stone using a continuous pulse, followed by a wash with normal saline to allow the broken stones to flow out of the body.

U100 laser lithotripsy was performed using the U100Plus laser lithotripter (Germany). The optical fiber of the laser was inserted into the bile duct through the choledochoscope. The laser capacity was set to 160mj and aligned with the center of the stone. After the stone was broken up, there was a rinse with saline to clear small stones, or they were taken out with a blue net. After the stones were removed, a drainage tube was inserted into the fistula and the puncture point was treated.

### Operation Indexes

The operation time, intraoperative bleeding, postoperative pain duration and hospital stay were recorded. Biliary bleeding and residual stones in the two groups were counted. The criteria for judging biliary bleeding were the presence of bloody fluid, detected by choledochoscopy, and the outflow of bloody fluid from the drainage tube.^9^ The criteria for residual stones were ultrasound and/or CT examination seven days after the operation.^10^ To assess liver function fasting venous blood samples were collected from 4l patients before the operation and seven days after the operation, basic patient information was recorded. Total bilirubin (TBIL), alanine aminotransferase (ALT) and albumin (ALB) levels were detected by enzyme-linked immunosorbent assay.

### Statistical Analysis

SPSS 22.0 was used for data processing, [n (%)] was used to represent the counting data for *χ^2^* test;(*X̅*±S) was used to represent measurement data for t-test;(P<0.05) was considered statistically significant.

## RESULTS

A total of 97 patients met the inclusion criteria of this retrospective study, including 51 males and 46 females. Their age ranged from 46 to 71 years, with an average of 59.08±6.89 years. Patients that were treated with pneumatic ballistic lithotripsy combined with PTCSL comprised Group-I, and 54 patients that were treated with U100 laser lithotripsy combined with PTCSL comprised Group-II.

There was no significant difference in the basic clinical data and in the operation time and intraoperative bleeding between the two groups (Table-I, P>0.05). The postoperative pain duration and hospital stay of patients in Group-II were shorter than those in Group-I (Table-II, P<0.05). Patients in Group-II had lower biliary bleeding rate (3.70% vs. 16.27%, P<0.05) and lower stone residue rate (1.85% vs. 11.63%, P<0.05) than patients in Group-I (Table-III). Before the operation, there was no significant difference in TBIL, ALT or ALB concentrations between the two groups (P>0.05). At 7 days post operation, both groups showed lower TBIL, ALT and ALB concentrations than what was observed before the operation. However, patients in Group-II had significantly higher levels of TBIL, ALT and ALB compared to Group-I patients (Table-IV, P<0.05).

**Table-I:** Comparison of patient characteristics between the two groups [n (%),*X̅*±S].

Group	n	Gender(male/Female)	Age(year)	Stone location(n)					

				Left intrahepatic bile duct	Right intrahepatic bile duct	Common bile duct	Left hepatolithiasis with common bile duct	Right hepatolithiasis with common bile duct	Intrahepatic bile duct
Group-I	43	22/21	59.35±7.20	10	13	7	6	4	3
Group-II	54	29/25	58.87±6.69	15	14	6	7	7	5
Χ^2^/t	-	0.062	0.338	1.278					
P	-	0.803	0.736	0.937					

**Table-II:** Comparison of surgical indicators between the two groups (*X̅*±S).

Group(n)	Operation time(min)	Intraoperative blood loss(ml)	Duration of postoperative pain(d)	Hospital stays(d)
Group-I(n=43)	160.09±20.04	179.93±18.35	4.28±1.12	10.74±2.05
Group-II(n=54)	162.48±21.40	182.42±16.90	2.30±0.72	7.17±1.79
t	0.338	0.562	10.082	9.176
P	0.736	0.576	<0.001	<0.001

**Table-III:** Comparison of biliary bleeding and residual stones in the two groups [n (%)].

Group	n	Biliary bleeding	Stone residue
Group-I	43	7(16.27)	5(11.63)
Group-II	54	2(3.70)	1(1.85)
x^2^	-	4.497	3.943
P		0.034	0.047

**Table-IV:** Comparison of liver function indexes between the two groups before operation and seven days after operation (*X̅* ±S).

	TBil(μmol/L)		t	P	ALT(U/L)		t	P	ALB(g/L)		t	P
*Group(n)*	Preoperative	7d after operation			Preoperative	7d after operation			Preoperative	7d after operation		
Group-I(n=43)	28.05±3.48	15.28±2.76	72.717	<0.001	71.14±4.49	42.30±4.07	262.109	<0.001	36.30±3.66	28.30±3.36	80.133	<0.001
Group-II(n=54)	28.54±3.61	19.33±2.84	58.534	<0.001	72.50±5.39	53.48±4.84	142.497	<0.001	36.57±4.22	32.68±3.85	24.986	<0.001
t	0.676	7.066	-	-	1.354	12.342	-	-	0.339	5.882	-	-

## DISCUSSION

Our study demonstrated that U100 laser and pneumatic trajectory combined with percutaneous transhepatic cholangiolithotomy is associated with shorter duration of pain and hospital stay in patients with intra-and extrahepatic bile duct stones. The rate of bile duct bleeding and stone residue in this group of patients was lower, which is similar to results by Lamanna A et al.^11^ Chunlin Y et al. explored the safety and effectiveness of ureteroscopic holmium laser lithotripsy (UHLL) and ureteroscopic pneumatic lithotripsy (UPL) in the treatment of impacted ureteral calculi (IUC). They showed that UHLL and UPL were safe and effective, but UHLL has the advantages of shorter operation time and high stone-free ratio.^12^ The mechanism of U100 laser lithotripsy combined with PTCSL uses a dual frequency and dual pulse Nd doped yttrium aluminum garnet laser, which can emit infrared light (wavelength 1064nm) and green light (wavelength 532nm). During lithotripsy, the stones absorb 20% green light to form uniform plasma, and the plasma has the ability to absorb 80% infrared light This promotes the conversion of laser energy into a mechanical flushing wave and disintegrates stones from the center.^13^ For example, Garg s et al. compared the efficacy, safety and complications of laser (Ho: YAG) and pneumatic ballistic internal lithotripsy in the treatment of ureteral calculi and showed that the stone clearance rate of laser lithotripsy was higher, it was safe and was not associated with complications.^14^ The driving force of the mechanical shock wave on the stones is lower than that used in pneumatic ballistic lithotripsy, which can reduce the damage to biliary tract, reduce postoperative pain and the biliary bleeding rate.

During lithotripsy, the stones can be pushed, which can damage the biliary wall, possibly causing severe postoperative pain and increasing the risk of biliary bleeding.^15^ In terms of stone removal, the ui000 light of U100 laser lithotripsy can be bent arbitrarily to enter the bile duct which can break and remove the more difficult stones, reducing incidences of any residual stones. In comparison, the pneumatic ballistic lithotripsy rod cannot be bent, which may cause the stones to shift and drift back, making it difficult to remove hidden or small stones, increasing the possibility of stone residue.^16^

When patients with intra-and extrahepatic bile duct stones are treated using surgery, the surgical trauma may cause damage to the patient’s liver and reduce their liver function.^17^ Therefore, in recent years, the protection of liver function is considered when designing and selecting the surgical procedure used for intra-and extrahepatic bile duct stone removal. In this study, TBIL, ALT and ALB concentrations within the two groups were significantly lower after the surgery. However, patients in Group-II, showed higher levels of TBIL, ALT and ALB than those in Group-I, post-operation. Muglia et al. compared and analyzed the effects of pneumatic ballistic lithotripsy and U100 laser lithotripsy on patients’ liver function during PTCSL.^18^ They found than patients treated with U100 laser lithotripsy had less postoperative liver function damage, consistent with the results of this study. It is suggested that U100 laser lithotripsy combined with PTCSL can reduce the negative impact on liver function when treating intra-and extrahepatic bile duct stones. The absorption coefficients of infrared light and green laser emitted by U100 laser lithotripsy do not cause a thermal effect during lithotripsy, and human tissues cannot absorb these two lasers, which eliminates the damage caused by the mechanical shock wave on the bile duct and liver, thereby effectively reducing the adverse impact on liver function.^19^ On the other hand, the process of pneumatic ballistic lithotripsy effectively pushes the projectile using compressed gas through lithotripsy probe rod, which can cause significant mechanical damage to the function of the biliary tract and liver.

### Limitations

The sample size was relatively small with only 97 patients who qualified for this study within our hospital. Additionally, patients were only monitored for seven days post-operation, which may make the conclusions one-sided and limited.

## CONCLUSION

U100 laser lithotripsy combined with PTCSL is more effective in the treatment of intra-and extrahepatic bile duct stones as compared to pneumatic ballistic combined with PTCSL. It is associated with lower postoperative pain, reduced incidence of biliary bleeding and stone residue, and significantly lesser negative impact on liver function.

### Authors’ contributions:

**ZY:** Conceived and designed the study.

**ZY & BS:** Collected the data and performed the analysis.

**ZY:** Was involved in the writing of the manuscript and is responsible for the integrity of the study.

All authors have read and approved the final manuscript.
